# Comparative Analysis of Functional and Structural Decline in Retinitis Pigmentosas

**DOI:** 10.3390/ijms21082730

**Published:** 2020-04-15

**Authors:** Thiago Cabral, Jose Ronaldo Lima de Carvalho, Joonpyo Kim, Jin Kyun Oh, Sarah R. Levi, Karen Sophia Park, Jimmy K. Duong, Junhyung Park, Katherine Boudreault, Rubens Belfort, Stephen H. Tsang

**Affiliations:** 1 Department of Ophthalmology, Jonas Children’s Vision Care and Bernard & Shirlee Brown Glaucoma Laboratory, Columbia University, New York, NY 10032, USA; thiagogeorge@hotmail.com (T.C.); jl5127@cumc.columbia.edu (J.R.L.d.C.J.); jo2532@cumc.columbia.edu (J.K.O.); srl2183@cumc.columbia.edu (S.R.L.); ksp2117@columbia.edu (K.S.P.); katherine.boudreault@umontreal.ca (K.B.); 2 Department of Specialized Medicine, CCS and Vision Center Unit, Ophthalmology, EBSERH/HUCAM, CCS-UFES—Federal University of Espírito Santo (UFES), Vitória, ES 29047-105, Brazil; 3 Department of Ophthalmology, Federal University of São Paulo (UNIFESP), São Paulo, SP 04039-032, Brazil; 4 Department of Ophthalmology, Empresa Brasileira de Serviços Hospitalares (EBSERH)–Hospital das Clínicas de Pernambuco (HCPE), Federal University of Pernambuco (UFPE), Recife, PE 50740-465, Brazil; 5 Department of Statistics, Seoul National University, Seoul 08826, Korea; joonpyokim@snu.ac.kr; 6 College of Medicine, State University of New York at Downstate Medical Center, Brooklyn, NY 11203, USA; 7 Department of Biostatistics, Columbia University, New York, NY 10032, USA; jkd2108@cumc.columbia.edu; 8 Department of Statistics, University of California, Los Angeles (UCLA), Los Angeles, CA 90095, USA; junhyung@ucla.edu; 9 Department of Pathology & Cell Biology, Stem Cell Initiative (CSCI), Institute of Human Nutrition, Vagelos College of Physicians and Surgeons, New York, NY 10032, USA

**Keywords:** retinitis pigmentosa, ERG, inherited retinal dystrophy

## Abstract

Retinitis pigmentosa (RP) is a category of inherited retinal dystrophies that is best prognosticated using electroretinography (ERG). In this retrospective cohort study of 25 patients with RP, we evaluated the correlation between 30 Hz flicker ERG and structural parameters in the retina. Internationally standardized 30 Hz flicker ERG recordings, short-wavelength autofluorescence (SW-AF), and spectral domain–optical coherence tomography (SD-OCT) were acquired at two visits at least one year apart. Vertical and horizontal hyperautofluorescent ring diameter measurements with SW-AF, as well as ellipsoid zone (EZ) line width measurements with SD-OCT, were used as structural parameters of disease progression. The 30 Hz flicker ERG amplitude decreased by 2.2 ± 0.8 µV/year (*p* = 0.011), while implicit times remained unchanged. For SD-OCT, the EZ line decreased by 204.1 ± 34.7 µm/year (*p* < 0.001). Horizontal and vertical hyperautofluorescent ring diameters decreased by 161.9 ± 25.6 µm/year and 146.9 ± 34.6 µm/year, respectively (*p* = 0.001), with SW-AF. A correlation was found between the progression rates of the 30 Hz flicker amplitude recorded with Burian–Allen electrodes and both the horizontal ring diameter (*p* = 0.020) and EZ line (*p* = 0.044). SW-AF and SD-OCT, two readily available imaging techniques, may be used to prognosticate disease progression because of the reliability of their measurements and correlation with functional outcome.

## 1. Introduction

Retinitis pigmentosa (RP) encompasses a group of inherited retinal dystrophies characterized by progressive visual field constriction, night blindness, and, in many cases, severe visual impairment at late disease stages [1,2]. Approximately 1 in 4000 individuals carry autosomal-dominant, autosomal-recessive, or X-linked mutations in any one of over 60 genes thus far discovered to cause RP [3]. The disease most commonly manifests as a rod–cone dystrophy, in which cone cell death occurs secondary to rod cell death [1]. In light of emerging gene therapy trials that may enable the development of FDA-approved RP treatments, such as voretigene neparvovec (Luxturna™) for *RPE65*-RP, it is imperative to establish reliable outcome measurements that can be used to monitor RP disease progression [4,5]. Current clinical practices for monitoring disease progression include the use of non-invasive imaging techniques and electroretinogram (ERG) testing.

Most clinicians employ non-invasive imaging techniques, such as short-wavelength fundus autofluorescence (SW-AF) and spectral domain–optical coherence tomography (SD-OCT), to aid in the diagnosis and monitoring of RP. SW-AF measures autofluorescence signals produced via the excitation of the bisretinoids formed in the outer segments of photoreceptors and later accumulated in the lipofuscin content of retinal pigment epithelium (RPE). In RP patients, SW-AF often reveals a ring of hyperautofluorescence considered to be a hallmark of the disease [6,7,8]. The size of this hyperautofluorescent ring was shown to progressively constrict and correlate with visual function, as measured by pattern ERG, and ellipsoid zone (EZ) line width, as seen with SD-OCT [9,10,11].

The relatively wide availability of these imaging techniques has led to their prominence in the clinical setting as the traditional tools used to evaluate patients with RP. A much less common but arguably more objective tool for characterizing RP disease is the ERG, which assesses the electrophysiological activity of the retina by administering light stimuli under dark-adapted (scotopic) or light-adapted (photopic) conditions [12]. The retinal function of patients with RP may be characterized by scotopic B-wave amplitudes and 30 Hz flicker ERG, the latter of which has been found to correlated with patients’ visual function in daily routine activities [13,14]. Early-stage RP may also be detected on ERG prior to the formation of any discernible disease markers in retinal imaging [12]. As such, ERG serves as a useful tool for diagnosing RP and determining the prognosis and trajectory of RP disease progression in patients [12]. 

Previous studies by our group characterized the natural disease history of RP by measuring the progressive decrease in EZ line width and constriction of the hyperautofluorescent ring diameters, as seen with SD-OCT and SW-AF imaging, respectively [15,16,17]. To determine whether the advantages of SW-AF and SD-OCT may be pooled with those of ERG, we assessed whether there were significant correlations between the disease progression rates derived from retinal imaging and those derived from parameters of visual function in ERG. 

## 2. Results

### 2.1. Patients

Twenty-five patients diagnosed with RP were included in the present study. The average age of the patients was 40.2 ± 4.3 years at the baseline visit and 43.0 ± 4.3 years at the follow-up visit, ranging from 12 to 80 years old. Eighteen patients had autosomal recessive RP; five had autosomal dominant RP; and two had X-linked RP. The mean follow-up time was 2.81 ± 0.55 years. Test–retest reliability measured by the intraclass correlation coefficient (ICC) presented high similarity for all parameters, namely EZ line (ICC > 0.99), horizontal ring diameter (ICC = 0.992), and vertical ring diameter (ICC = 0.972) measurements. Demographic, clinical, and genetic data are summarized in Table 1, Table 2 and Table 3.

Progression of 30 Hz flicker amplitudes was measured by Dawson–Trick–Litzkow (DTL) fiber or Burian–Allen (BA) corneal contact lens electrodes. Fourteen patients underwent ERG testing with DTL electrodes, while 11 patients underwent testing with BA electrodes. Interestingly, the mean 30 Hz flicker amplitudes acquired using DTL at visits 1 and 2 were approximately 5 and 12 times greater, respectively, than the mean amplitudes acquired with BA electrodes (Table 4). This can be explained by the fact that BA electrode use was generally reserved for patients with severe RP, which requires tighter noise control to yield detectable ERG signals. However, comparative statistics using a Welch’s *t*-test did not reveal a statistically significant difference between either methods of ERG recording (*p* = 0.121). 

The mean amplitude of the 30 Hz flicker ERG among all 25 patients was 23.4 ± 5.72 µV at the baseline visit and 18.4 ± 5.5 µV at follow-up, while the mean 30 Hz implicit time was 34.0 ± 1.0 ms at baseline and 34.2 ± 0.9 ms at follow-up. The mean rate of disease progression as measured by the 30 Hz flicker amplitude was −2.2 ± 0.8 µV/year (*p* = 0.011), while no significant progression was detected in the 30 Hz implicit time (Table 5). 

### 2.2. Correlations between the Progression Rates of Structural and Functional Parameters in the Retina

**Figure 1:**
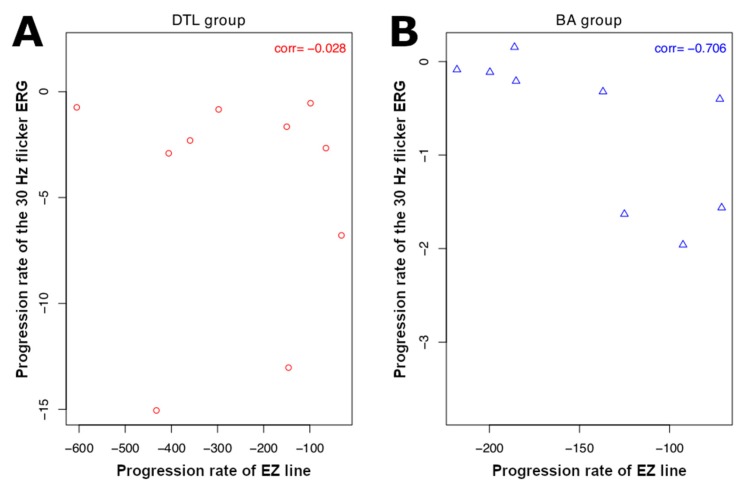
Scatter plots of the progression rate of 30 Hz flicker electroretinogram (ERG) amplitude versus that of ellipsoid zone (EZ) lines: (**A**) progression rates of patients in DTL and (**B**) BA groups. Correlation between progression rates is not significant in DTL group, while in BA group significant negative correlation (−0.706) is observed. Here ’corr’ at the top-right corner of each subplot denotes correlation coefficient

Among 20 patients (40 eyes) with available SD-OCT images, two eyes of one patient exhibiting an irregular and broken EZ line and one eye with an EZ extending beyond the SD-OCT line scan area were excluded from analysis. The mean EZ line width was 3193.4 ± 419.3 µm at the baseline visit and 2730.4 ± 367.7 µm at follow-up, with a mean progression rate of −204.1 ± 34.7 µm/year (*p* < 0.001) (Table 5; see Table S1 for stratification by group). While no significant correlation was observed between the rate of progression of the ERG measurements and that of the EZ line in the DTL group, a correlation of −0.71 was found between these same parameters for the patients who underwent ERG with BA electrodes (*p* < 0.05) (Table 6). The relationship between the rate of progression of the 30 Hz flicker ERG and that of the EZ line in both DTL and BA groups is displayed in Figure 1. 

**Figure 2:**
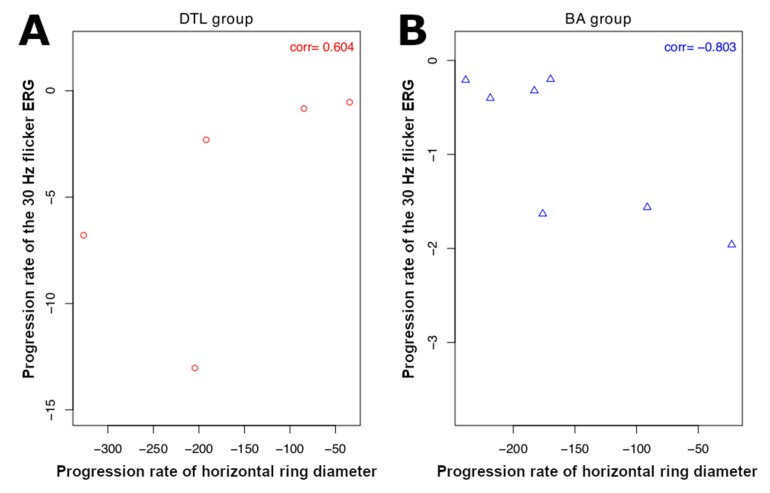
Scatter plot of the progression rate of 30 Hz flicker electroretinogram (ERG) amplitude versus that of horizontal ring diameter: (**A**) progression rates of patients in DTL and (**B**) BA groups. Correlation between progression rates is not significant in DTL group, while in BA group significant negative correlation (−0.803) is observed. Here ’corr’ at the top-right corner of each subplot denotes correlation coefficient.

**Figure 3:**
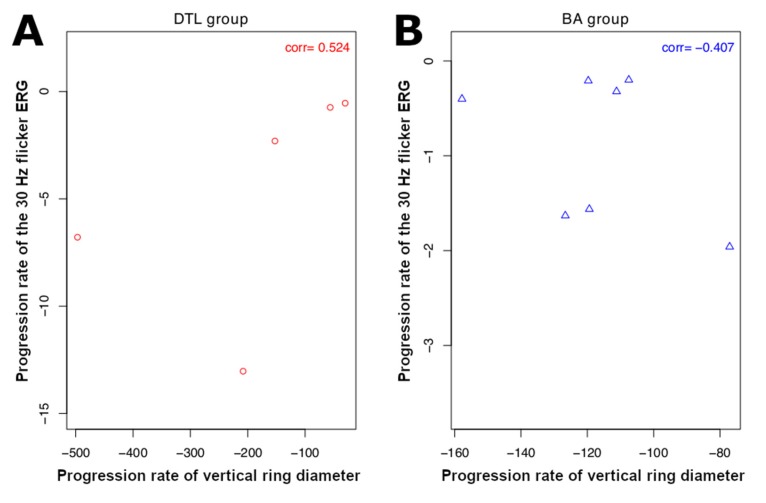
Scatter plots of the progression rate of 30 Hz flicker electroretinogram (ERG) amplitude versus that of vertical ring diameter: (**A**) progression rates of patients in DTL and (**B**) BA groups. Correlations between progression rates are not significant in either DTL or BA groups. Here ’corr’ at the top-right corner of each subplot denotes correlation coefficient.

SW-AF images were available for 20 patients (40 eyes) at the baseline visit. Among these, a hyperautofluorescent ring was visible in 23 eyes of 15 patients, while 17 eyes did not show a ring and were excluded from analysis. Three additional eyes were also excluded due to the size of the ring extending beyond the boundaries of the 30° × 30° field image. The mean horizontal diameter of the ring was 3567.7 ± 483.6 µm at baseline and 3195.2 ± 463.2 µm at follow-up, with a mean progression rate of −161.9 ± 25.6 µm/year (*p* < 0.001). For the vertical ring diameter, the mean value was 3136.8 ± 486.7 µm at baseline and 2812.7 ± 465.7 µm at follow-up, with a mean progression rate of −146.9 ± 34.6 µm/year (*p* = 0.001) (Table 5, see Table S1 for stratification by group). Similarly, no significant correlation was observed between the rates of progression of the horizontal and vertical ring diameters and the ERG measurements when the DTL group was analyzed (Table 7 and Table 8). However, a significant correlation of −0.80 was found between the rates of progression of the horizontal ring diameter and the 30 Hz flicker amplitude for the patients who had ERG recorded with BA electrodes (*p* = 0.020) (Table 7), while no association was found between progression of the vertical ring diameter and ERG measurements (Table 8). The rates of progression of the 30 Hz flicker ERG, horizontal, and vertical ring diameters of available patients are displayed in Figure 2 and Figure 3. 

## 3. Discussion

Our study evaluated the correlations between the progression rates derived from ERG and structural imaging techniques in patients with RP. ERG has been used as the gold standard for prognosticating and predicting remaining vision lifetime in patients with RP [13]. While ERG serves as a useful parameter as it measures the dynamic functionality of the photoreceptor cells rather than their static anatomy, it has disadvantages due to high test–retest variability [18], along with the fact that ERG machines are not as widely available in most retina services. Moreover, the clinical implementation of ERG requires a well-trained team of specialists capable of performing the test properly and analyzing the results. As such, the use of a more practical and easily accessible clinical tool that conveys similar prognostic information would be helpful for services caring for patients with RP. SD-OCT and SW-AF measurements have been shown to be reproducible, with very strong test–retest correlation (ICC > 0.97 for all parameters), and are correlated with functional outcomes.

Among the severe patients with RP for whom ERG was measured by BA electrodes, in our data we observed significant negative correlations between the disease progression rates of the horizontal hyperautofluorescent ring measured by SW-AF, the EZ line width measured by SD-OCT, and the amplitude of the 30 Hz flicker ERG. However, no correlation was identified for the patient group who received ERG with DTL electrodes. This may be explained by the greater variability in the ERG signal captured with DTL [19,20] as opposed to BA electrodes. Moreover, we reserved the use of BA electrodes for more severe patients and the use of DTL electrodes for milder patients, as the latter option is more patient friendly. Because of this, severe patients who underwent ERG testing with BA electrodes had a small EZ line width and an autofluorescent ring localized at the fovea. The fovea is populated only by cones, which are the source of the 30 Hz flicker ERG [21]. Thus, a lack of correlation for the milder group measured by DTL, as opposed to the severe group measured by BA, may also be explained by the fact that, due to the stage of disease, structural imaging for the DTL group was representative of rods and cones, while for the BA group this was mostly representative of cones. As RP is a rod–cone dystrophy, rod loss occurs before cone loss [22]. The EZ line width and autofluorescent ring constriction in the DTL group predominantly represented rod death, while in the BA group those same parameters predominantly represented cone death. In consequence, changes to the latter measurements likely had a greater impact on the 30 Hz ERG signal.

Previous studies have demonstrated that the area encompassed by the hyperautofluorescent ring and the EZ line represents the area of the retina that remains functional [7,9,10,11]. Under this premise, we expected a correlation to be present between the rate of constriction of the ring diameters and the rate of decrease of the 30 Hz flicker amplitude, given that the ERG measures the electrophysiological activity of those same cells visible with SW-AF and SD-OCT.

**Figure 4:**
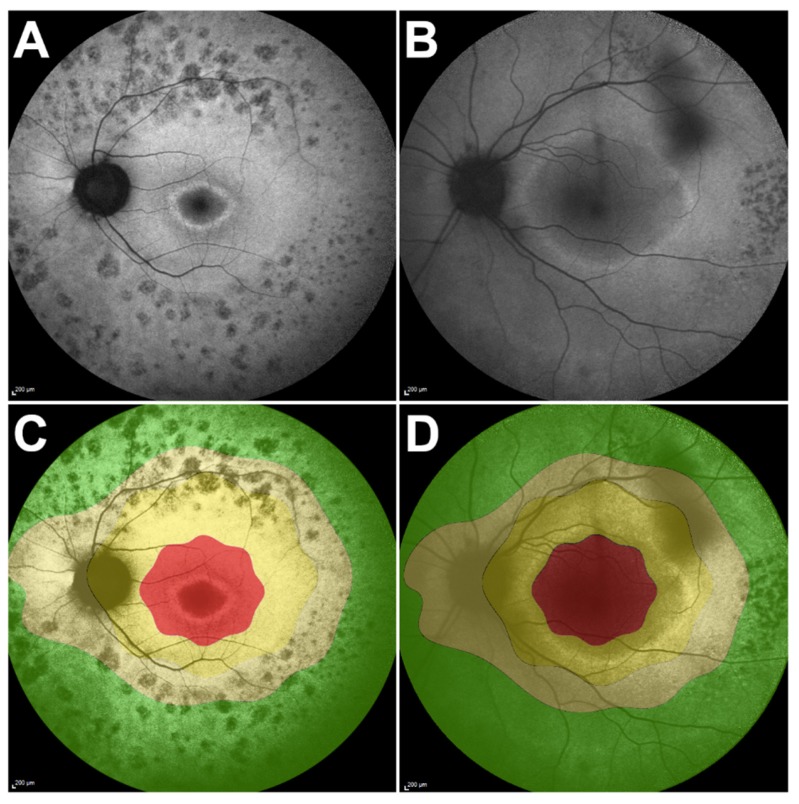
Retintis pigmentosa autofluorescent ring and its correlation with cone photoreceptor cells in the retina. (**A**,**B**) Autofluorescence images of P18 and P22, respectively. Note the position of the autofluorescent ring and its relation to cone photoreceptor cell distribution (**C**,**D**). Red, high cone density; yellow, medium cone density; green, low cone density. In P18 (**C**), the ring falls in the high cone density zone (red), as opposed to the medium density zone (yellow) in P22. As such, the P18 ring constriction has more impact in the 30 Hz flicker electroretinogram response than the similar constriction for P22.

**Figure 5:**
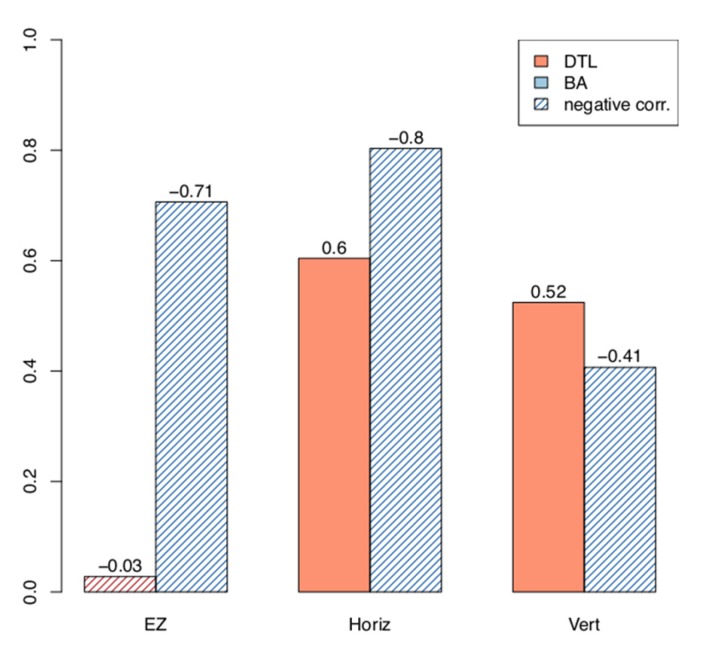
Correlations between progression rates of 30 Hz flicker electroretinogram (ERG) amplitude and structural parameters. Red bars represent correlations in the DTL group, while blue bars represent those in the BA group. Bars with shaded lines denote the negative correlations. ‘EZ’, ‘Horiz’, and ‘Vert’ denote the ellipsoid zone line width, the horizontal ring diameter, and the vertical ring diameter, respectively.

Previous imaging studies have shown that RP progresses in an exponential fashion [15,17,23]. In other words, a fast rate of progression is seen when the disease advances from the far periphery towards the center, while a slow rate of progression is seen once the disease reaches the macular region. However, in terms of ERG, since the cone photoreceptors, which are responsible for the 30 Hz flicker ERG signal, are more populated in the macula [21], a larger drop in the amplitude of ERG relative to the baseline is expected when the central cells are affected, as opposed to the loss of peripheral retina (Figure 4). Thus, a negative correlation is in agreement with our expectations. Conversely, no significant correlation was observed between the progression rate of ERG and that of the vertical ring diameter (Figure 5).

It is known that the hyperautofluorescent ring manifests as an ellipsoid in the early stages of RP [10], which would imply that there are differences in the lengths of the vertical and horizontal diameters when the disease first becomes evident with SW-AF. Studies using adaptive optics flood illumination have revealed histological and anatomical differences in the photoreceptor distribution between both vertical and horizontal meridians, which may explain the ellipsoid shape of the ring [24,25]. Nevertheless, this ellipsoid has been observed to progressively evolve into a uniformly round shape, which occurs due to a difference in the rate of one ring diameter’s constriction compared to that of the other diameter. In our study, we found a faster rate of progression for the horizontal diameter in comparison to the vertical (Table 5), which is in accordance with the findings in the literature [26,27]. Thus, it is expected that the horizontal diameter would have a better correlation with ERG as opposed to the vertical diameter of the hyperfluorescent ring, due to the faster progression of the former. As such, we believe that a longer follow-up may show a correlation between the vertical diameter and 30 Hz flicker ERG.

To our knowledge, this is the first study that correlates rates of RP progression derived from ERG with those of structural imaging techniques, namely SW-AF and SD-OCT. Our study suggests that SW-AF and SD-OCT, two reliable and widely available imaging techniques, may be used in lieu of 30 Hz flicker ERG to prognosticate disease in patients with RP. Moreover, test–retest reliability was shown to be less variable for SD-OCT and SW-AF than for ERG. Of note, the retrospective design and small number of subjects are important limitations to our study. Results that we observed need to be replicated in other studies by ourselves and other groups. Furthermore, the heterogeneity of our patient population, characterized by the broad age range and spectrum of RP disease, may have skewed the mean rates of disease progression, consequently resulting in inaccurate correlations; due to the logarithmic progression of RP disease in patients, subjects at the initial stage of disease may exhibit a faster rate of progression than those at the end stage of disease [28]. Future RP progression studies that stratify the patient cohort by age range and severity of disease utilizing a larger sample size of patients would, thus, provide a more accurate depiction of disease that better resembles a logarithmic rate of progression.

## 4. Materials and Methods

### 4.1. Subjects and Clinical Examination

A retrospective analysis of 25 patients diagnosed with RP was performed. A waiver of some or all elements of informed consent was granted. All study procedures were defined as outlined by protocol #AAAR8743 and approved by the Institutional Review Board at Columbia University (approved on 20 December 2019). The study adhered to the tenets of the Declaration of Helsinki. All patients presented to the Department of Ophthalmology at Edward S. Harkness Eye Institute, Columbia University. The inclusion criterion for this study was a clinical diagnosis of bilateral RP. In addition, each patient was screened for a history of two visits in our office at least one year apart, consisting of a complete ophthalmic examination by a retinal physician (S.H.T.). Ophthalmic examinations included a slit-lamp and dilated funduscopic examination, SW-AF (488 nm excitation) and SD-OCT imaging, and full-field electroretinogram (ffERG) testing. The diagnosis of RP was made based on presenting symptoms, namely night blindness and visual field restriction, fundus appearance, family history, and ffERG results. Internationally standardized 30 Hz flicker ERG recordings were acquired using DTL fiber or BA corneal contact lenses. None of the data presented in this study, including images and genetic testing results, are identifiable to individual patients.

### 4.2. Genotyping

DNA was obtained from peripheral blood of the affected individuals and molecular diagnosis was performed. A next generation sequencing (NGS) retinal dystrophy panel was performed for patients 4, 14, and 16 at the Molecular Vision Lab in Hillsboro, Oregon, and for patients 15 and 25 at the Prevention Genetics laboratory in Marshfield, Wisconsin. Patients 5, 9, and 17 underwent clinical whole exome sequencing with Agilent SureSelectXT Human All Exon V5 + untranslated regions (UTR) capture and Illumina HiSeq2500 sequencing technology. The detection and analysis of pathogenic mutations was completed using the NextGENE software from Softgenetics and the proprietary analytical pipeline of the Personalized Genomic Medicine (PGM) laboratory at Columbia University. Patient 11 underwent retinal panel testing in the same laboratory. Patients 7, 8, and 20 were tested through the National Ophthalmic Disease Genotyping Network (eyeGENE) and direct sequencing of the target gene was performed. Single nucleotide polymorphism (SNP) array allowing for detection of loss of copy number variants (deletion) was done for patient 10. Patient 19 underwent whole exome sequencing at Ambry Genetics, and patients 6, 18, and 23 had Sanger sequencing performed in our research laboratory. Lastly, patients 1, 2, 3, 12, 13, 18, 21, 22, and 24 did not receive genetic testing.

### 4.3. Electrophysiology

Full-field electroretinograms (Diagnosys LLC, Lowell, Massachusetts, USA) were recorded from both eyes using DTL fiber or BA corneal contact lens electrodes according to the standards from the International Society for Clinical Electrophysiology of Vision (ISCEV) in both scotopic and photopic states [27]. The amplitudes and implicit times obtained from both eyes of each patient were compared with age-matched control values.

### 4.4. Image Acquisition and Measurements

SW-AF (488 nm excitation, barrier filter transmitted light from 500 to 680 nm, 30° × 30° field) and SD-OCT images were acquired using a confocal scanning laser ophthalmoscope (cSLO; Spectralis HRA + OCT, Heidelberg Engineering, Heidelberg, Germany). Pupils were dilated to at least 7 mm in diameter using 1% tropicamide and 2.5% phenylephrine before image acquisition. SD-OCT images were taken as horizontal 9 × 9 mm scans (870 nm; 7 µM axial resolution) through the macula acquired in high-resolution mode with averaging of 100 single scans. The scans were registered automatically to a simultaneously acquired infrared reflectance (IR-R) (820 nm) fundus image. The nomenclature used to identify reflectivity bands in SD-OCT images was as published [29].

**Figure 6:**
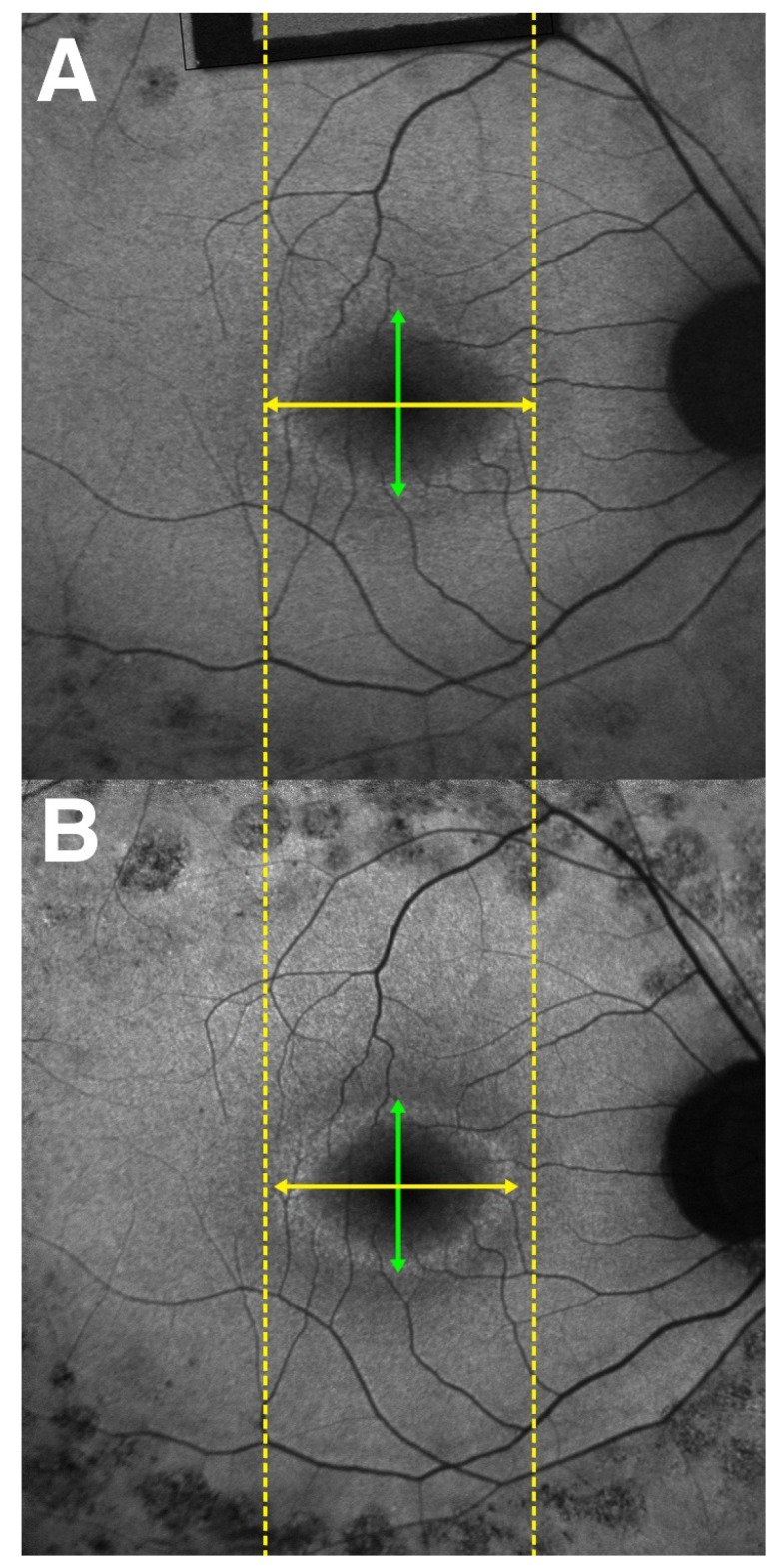
Progression of hyperautofluorescent ring diameters seen in short-wavelength autofluorescence (SW-AF) imaging of a patient with retinitis pigmentosa (RP). (**A**) A SW-AF scan of a hyperautofluorescent ring in RP at the baseline visit. Dashed yellow lines are tangential to the horizontal-most edges of the hyperautofluorescent ring. Green lines span the vertical diameter of the ring, while yellow lines span the horizontal diameter. The horizontal diameter of the ring is noticeably larger at the baseline visit than at the follow-up (**B**).

**Figure 7:**
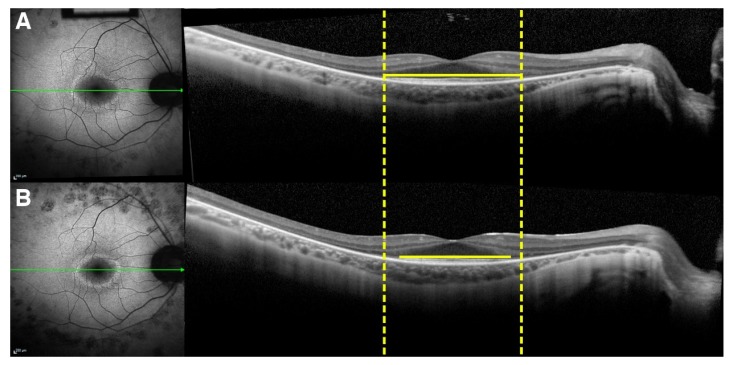
Ellipsoid zone (EZ) line progression in spectral domain–optical coherence tomography (SD-OCT) imaging of a patient with retinitis pigmentosa (RP). Green lines represent the position of the horizontal OCT scan with respect to the autofluorescence image. Dashed yellow lines are tangential to the lateral ends of the EZ line shown in the horizontal SD-OCT cross-section through the fovea at the baseline visit (**A**). The EZ line width, indicated by the span of the yellow lines, is greater at the baseline visit than at follow-up (**B**).

The external boundary of the hyperautofluorescent ring was manually measured by its horizontal and vertical diameters using the measuring tool provided in the Spectralis software (Figure 6). In cases in which a complete ring was not seen, only the observable axis was measured. The external edge of the ring was preferred over the internal edge owing to its greater definition and distinct appearance, allowing for more precise measurements. The interest in studying photoreceptor survival led to the decision to measure the EZ line width due its reliability, as opposed to the interdigitation zone (IZ) line, which cannot always be distinguished, even in normal subjects [30]; the EZ line is readily detectable in every patient. The EZ width was manually measured on the high-resolution horizontal scan through the fovea using the measuring tool in the Spectralis software (Figure 7).

### 4.5. Statistical Analysis

Structural measurements were obtained from SD-OCT and SW-AF images, while 30 Hz flicker values were obtained from ERG performed at each visit. Measurements were obtained twice, two weeks apart, by the first author (T.C.). Test–retest reliability of the measurements for each parameter was assessed by calculating the intraclass correlation coefficient (ICC) using R version 3.61 (Vienna, Austria). The mean of the two measurements was used for analysis purposes. For each statistical analysis, the averages of the values obtained from both eyes of each patient (when available) were utilized. Progression rates, defined as the difference between the values obtained from the baseline and second visits divided by the follow-up time, were calculated for each parameter and compared to zero using a one sample Student’s *t*-test. The Pearson correlation coefficient was used to examine the relationships between the progression of structural (EZ line width and horizontal and vertical hyperautofluorsecent ring diameters) and functional (30 Hz flicker values) parameters. For the correlation, we report two *p*-values, with the standard one (Corr Test) assuming a bivariate normal distribution model and the other one taken from a permutation test (Perm Test) with at most 10,000 permutations. A Welch’s two sample *t*-test was performed to compare the progression of 30 Hz flicker values as measured by DTL versus BA corneal contact lens electrodes.

### 4.6. Data Availability Statement

The datasets generated and analyzed during the current study are available from the corresponding authors on reasonable request.

## Figures and Tables

**Table 1:** Demographic and genetic characterization of study patients.

Patient	Sex	Age	Diagnosis	Gene
1	F	80	ARRP	NA
2	M	73	ADRP	NA
3	F	75	ARRP	NA
4	M	40	ARRP	MAK 353bp ins Alu
5	F	37	ARRP	USH2A c.10073G>A (p.Cys3358Tyr) and c.920_923dupGCCA (p.His308Glnfs)
6	M	12	ADRP	RHO c.568G>A (p.Asp190Asn)
7	F	23	ARRP	CNGB1 c.3150del (p.Phe1051Leufs*12) homozygous
8	M	17	ARRP	CNGB1 c.3150del (p.Phe1051Leufs*12) homozygous
9	M	75	ARRP	WES negative
10	F	23	ARRP	MERTK homozygous partial deletion (two-copy loss 2q13)
11	F	49	XLRP	RPGR c.2203_2226del124 (p.His735_Glu742del)
12	F	43	ADRP	NA
13	F	26	ARRP	NA
14	M	32	ARRP	KIAA1549 c.358A>G (p.Thr120Ala) and c.1925C>T (p.Ser642Leu)
15	M	58	ARRP	USH2A c.12575G>A (p.Arg4192His) and c.7595-2144A>G (p.?)
16	F	16	ADRP	RHO c.937-27_-19delCCCTGACTC (p.?)
17	M	30	ARRP	WES negative
18	M	31	ARRP	NA
19	F	12	ARRP	MERTK c.2189+1G>T (p.?) homozygous
20	M	12	XLRP	RPGR c.202G>A (p.Gly68Arg)
21	M	38	ARRP	NA
22	M	58	ARRP	NA
23	M	26	ADRP	RP1 c.2029C>T (p.Arg667Ter)
24	F	50	ARRP	NA
25	M	69	ARRP	USH2A c.13378A>T (p.Ile4460Leu)

ADRP: autosomal dominant retinitis pigmentosa; ARRP: autosomal recessive retinitis pigmentosa; XLRP: X-linked retinitis pigmentosa; NA: not available. The asterisk denotes stop codon after the frameshift.

**Table 2:** Study subjects’ sex, mean age and standard deviation (SE), and follow-up times.

	N (%)	Mean Age at Baseline Visit (Years) (SE)	Mean Age at Follow-Up Visit (Years) (SE)	Min. Age at Presentation (Years)	Max. age at presentation (Years)	Mean Follow-Up Time (Years) (SE)
Patients	25	40.2 (4.3)	43.0 (4.3)	12	80	2.81 (0.55)
Male	14/25 (56)					
Female	11/25 (44)					

**Table 3:** Study subjects’ sex, mean age and standard deviation (SE), and follow-up times by subgroup.

	N (%)	Mean Age at Baseline Visit (Years) (SE)	Mean Age at Follow-Up Visit (Years) (SE)	Min. Age at Presentation (Years)	Max. Age at Presentation (Years)	Mean Follow-Up Time (Years) (SE)
Dawson–Trick–Litzkow (DTL) Patients	14	43.3 (5.7)	46.2 (5.7)	12	80	2.90 (0.5)
Male	7/14 (50)					
Female	7/14 (50)					
Burian–Allen (BA) Patients	11	36.3 (6.9)	39.0 (6.8)	12	75	2.69 (0.6)
Male	7/11 (64)					
Female	4/11 (36)					

**Table 4:** Comparison of 30 Hz Flicker electroretinogram (ERG) values recorded using Dawson–Trick–Litzkow (DTL) fiber versus Burian–Allen (BA) corneal contact lens electrodes. Data are summarized as mean and standard deviation (SE).

Parameter	N	30Hz Flicker ERG (µV) Baseline Mean (SE)	30Hz Flicker ERG (µV) Follow-Up Mean (SE)	Rate of Progression Mean (SE)	*p*-Value
Dawson–Trick–Litzkow (DTL) fiber	14	36.5 (8.6)	30.9 (8.4)	−3.22 (1.4)	0.121
Burian–Allen (BA) corneal contact lenses	11	6.7 (2.7)	2.6 (0.6)	−0.91 (0.4)	

**Table 5:** Rates of disease progression per year with respect to electroretinogram (ERG) and structural imaging parameters. Data are summarized as mean and standard deviation (SE).

Parameter	N	Baseline Visit Mean (SE)	Follow-Up Visit Mean (SE)	Rate of Progression Mean (SE)	*p*-Value
30 Hz Amplitude (µV)	25	23.4 (5.72)	18.4 (5.5)	−2.2 (0.8)	0.011
30 Hz Implicit Time (ms)	25	34 (1.0)	34.2 (0.9)	0.3 (0.5)	0.514
EZ line (µm)	19	3193.4 (419.3)	2730.4 (367.7)	−204.1 (34.7)	<0.001
Horizontal Ring Dimeter (µm)	12	3567.7 (483.6)	3195.2 (463.2)	−161.9 (25.6)	<0.001
Vertical Ring Diameter (µm)	12	3136.8 (486.7)	2812.7 (465.7)	−146.9 (34.6)	0.001

**Table 6:** Correlations between progression of 30 Hz flicker amplitude versus progression of ellipsoid zone (EZ) line width.

EZ Line Width	N	Correlation Coefficient	*p*-Value	
			Corr Test *	Perm Test **
DTL	10	−0.03	0.939	0.938
BA	9	−0.71	0.033	0.044

* ’Corr Test’ denotes the standard correlation test assuming a bivariate normal distribution model, and ** ‘Perm Test’ denotes the permutation test with at most 10,000 permutations.

**Table 7:** Correlations between progression of 30 Hz flicker amplitude versus progression of horizontal ring diameter.

Horizontal Ring Diameter	N	Correlation Coefficient	*p*-Value	
			Corr Test *	Perm Test **
DTL	5	0.60	0.280	0.276
BA	7	−0.80	0.030	0.020

* ’Corr Test’ denotes the standard correlation test assuming a bivariate normal distribution model, and ** ‘Perm Test’ denotes the permutation test with at most 10,000 permutations.

**Table 8:** Correlations between progression of 30 Hz flicker amplitude versus progression of vertical ring diameter.

Vertical Ring Diameter	N	Correlation Coefficient	*p*-Value	
			Corr Test *	Perm Test **
DTL	5	0.52	0.364	0.365
BA	7	−0.41	0.365	0.449

* ’Corr Test’ denotes the standard correlation test assuming a bivariate normal distribution model, and ** ‘Perm Test’ denotes the permutation test with at most 10,000 permutations.

## Supplementary Materials

The following are available online at https://www.mdpi.com/1422-0067/21/8/2730/s1.
