# Supplementary material for: Comparative Analysis of Functional and Structural Decline in Retinitis Pigmentosas
Source: Int J Mol Sci. 2020 Apr 15;21(8):2730. doi: 10.3390/ijms21082730 (PMC7215932; doi:10.3390/ijms21082730)
Supplement: Supplementary file 1 [file ijms-21-02730-s001.pdf]

Table S1. Rates of disease progression per year with respect to structural imaging parameters per group.

Parameter	N	Baseline Visit Mean (SE)	Follow-up Visit Mean (SE)	Rate of Progression Mean (SE)
**EZ line (µm)**				
BA	9	2323.4 (492.6)	2007.1 (436.5)	-143.0 (18.9)
DTL	10	3976.5 (574.5)	3381.4 (511.7)	-259.2 (59.9)
**Horizontal Ring Dimeter (µm)**				
BA	7	2594.5 (410.0)	2226.6 (386.1)	-157.2 (28.4)
DTL	5	4930.2 (628.3)	4551.2 (561.1)	-168.4 (50.9)
**Vertical Ring Diameter (µm)**				
BA	7	1944.3 (345.0)	1651.4 (300.7)	-117.0 (9.1)
DTL	5	4806.3 (345.2)	4438.6 (329.9)	-188.7 (83.5)
